# Calcineurin Inhibitor-Based Immunosuppression and COVID-19: Results from a Multidisciplinary Cohort of Patients in Northern Italy

**DOI:** 10.3390/microorganisms8070977

**Published:** 2020-06-30

**Authors:** Lorenzo Cavagna, Elena Seminari, Giovanni Zanframundo, Marilena Gregorini, Angela Di Matteo, Teresa Rampino, Carlomaurizio Montecucco, Stefano Pelenghi, Barbara Cattadori, Eleonora Francesca Pattonieri, Patrizio Vitulo, Alessandro Bertani, Gianluca Sambataro, Carlo Vancheri, Alessandro Biglia, Emanuele Bozzalla-Cassione, Valentina Bonetto, Maria Cristina Monti, Elena Ticozzelli, Annalisa Turco, Tiberio Oggionni, Angelo Corsico, Francesco Bertuccio, Valentina Zuccaro, Veronica Codullo, Monica Morosini, Carlo Marena, Massimiliano Gnecchi, Carlo Pellegrini, Federica Meloni

**Affiliations:** 1 Rheumatology Division, University of Pavia and IRCCS Policlinico S. Matteo Foundation of Pavia, 27100 Pavia, Italy; gio.zanframundo@gmail.com (G.Z.); c.montecucco@smatteo.pv.it (C.M.); alessandro.biglia@libero.it (A.B.); emanuele.bozzalla@gmail.com (E.B.-C.); veronicacodullo@yahoo.it (V.C.); 2 Infectious Diseases Clinic, University of Pavia and IRCCS Policlinico S. Matteo Foundation, 27100 Pavia, Italy; e.seminari@smatteo.pv.it (E.S.); a.dimatteo@smatteo.pv.it (A.D.M.); v.zuccaro@smatteo.pv.it (V.Z.); 3 Nephrology, Dialysis and Transplantation Unit, University of Pavia and IRCCS Policlinico S. Matteo Foundation of Pavia, 27100 Pavia, Italy; mgregorini@hotmail.com (M.G.); T.Rampino@smatteo.pv.it (T.R.); ef.pattonieri@gmail.com (E.F.P.); 4 Division of Cardiac Surgery, IRCCS Policlinico S. Matteo Foundation of Pavia, 27100 Pavia, Italy; s.pelenghi@smatteo.pv.it (S.P.); b.cattadori@smatteo.pv.it (B.C.); C.Pellegrini@smatteo.pv.it (C.P.); 5 Pulmonology Unit, IRCCS Istituto Mediterraneo Trapianti e Terapie ad Alta Specializzazione (ISMETT), 90100 Palermo, Italy; pvitulo@ismett.edu; 6 Thoracic Surgery Unit, IRCCS Istituto Mediterraneo Trapianti e Terapie ad Alta Specializzazione (ISMETT), 90100 Palermo, Italy; abertani@ismett.edu; 7 Regional Referral Centre for Rare Lung Diseases, A. O. U. “Policlinico-Vittorio Emanuele” Department of Clinical and Experimental Medicine, University of Catania, 95125 Catania, Italy; dottorsambataro@gmail.com (G.S.); vancheri@unict.it (C.V.); 8 Department of Biochemistry and Molecular Pharmacology, Istituto di Ricerche Farmacologiche Mario Negri IRCCS, 20156 Milan, Italy; valentina.bonetto@marionegri.it; 9 Department of Public Health, Unit of Biostatistics and Clinical Epidemiology, University of Pavia, 27100 Pavia, Italy; cristina.monti@unipv.it; 10 General Surgery Unit, IRCCS Policlinico S. Matteo Foundation of Pavia, 27100 Pavia, Italy; e.ticozzelli@smatteo.pv.it; 11 Cardiology Department, IRCCS Policlinico S. Matteo Foundation of Pavia, 27100 Pavia, Italy; A.Turco@smatteo.pv.it; 12 Department of Respiratory Diseases, University of Pavia and IRCCS Policlinico S. Matteo Foundation of Pavia, 27100 Pavia, Italy; t.oggionni@smatteo.pv.it (T.O.); corsico@unipv.it (A.C.); francesco.bertuccio01@gmail.com (F.B.); M.Morosini@smatteo.pv.it (M.M.); f.meloni@smatteo.pv.it (F.M.); 13 SC Direzione Medica di Presidio, IRCCS Policlinico S. Matteo Foundation of Pavia, 27100 Pavia, Italy; c.marena@smatteo.pv.it; 14 Coronary Care Unit and Laboratory of Clinical and Experimental Cardiology, Department of Medical Sciences and Infectious Disease, IRCCS Policlinico S. Matteo Foundation of Pavia, 27100 Pavia, Italy; massimiliano.gnecchi@unipv.it; 15 Department of Molecular Medicine, Cardiology Unit, University of Pavia, 27100 Pavia, Italy

**Keywords:** COVID-19, calcineurin inhibitors, solid organ transplantation, rheumatic diseases

## Abstract

The role of immunosuppression in SARS-CoV-2-related disease (COVID-19) is a matter of debate. We here describe the course and the outcome of COVID-19 in a cohort of patients undergoing treatment with calcineurin inhibitors. In this monocentric cohort study, data were collected from the COVID-19 outbreak in Italy up to 28 April 2020. Patients were followed at our hospital for solid organ transplantation or systemic rheumatic disorders (RMDs) and were on calcineurin inhibitor (CNI)-based therapy. Selected patients were referred from the North of Italy. The aim of our study was to evaluate the clinical course of COVID-19 in this setting. We evaluated 385 consecutive patients (220 males, 57%; median age 61 years, IQR 48–69); 331 (86%) received solid organ transplantation and 54 (14%) had a RMD. CNIs were the only immunosuppressant administered in 47 patients (12%). We identified 14 (4%) COVID-19 patients, all transplanted, mainly presenting with fever (86%) and diarrhea (71%). Twelve patients were hospitalized and two of them died, both with severe comorbidities. No patients developed acute respiratory distress syndrome or infectious complications. The surviving 10 patients are now fully recovered. The clinical course of COVID-19 patients on CNIs is generally mild, and the risk of superinfection seems low.

## 1. Introduction

Coronavirus disease 2019 (COVID-19) was first reported in China in 2019 and is related to a new strain of coronavirus, called “severe acute respiratory syndrome coronavirus-2” (SARS-CoV-2) [1]. Similarly to 2003 SARS-CoV and the “Middle East respiratory syndrome” (MERS) coronavirus [2], SARS-CoV-2 severely affects the lungs and COVID-19 patients are at increased risk of developing fatal acute respiratory distress syndrome (ARDS) [3]. From February 2020, COVID-19 affected regions in Northern Italy, causing an elevated number of hospital and intensive care unit (ICU) admissions [4]. According to data released by our National Institute of Health (Istituto Superiore di Sanità—ISS) [5] on April 28, 2020, the prevalence of COVID-19 infection in Veneto, Emilia-Romagna, Piedmont, and Lombardy was between 361 and 739 patients/100,000 inhabitants. Fifteen percent of patients had severe clinical presentation requiring hospitalization, and three percent needed admission to an intensive care unit (ICU). One unresolved issue about COVID-19 is the influence of immunosuppressive treatments (ISTs) on disease occurrence and course. The results published so far are rather contradictory, with authors reporting either mild [6,7,8,9,10,11,12] or severe disease course with high mortality [13,14,15,16,17,18]. These differences could be due to the different approaches adopted for therapy management (continuation or interruption of ISTs). Another confusing factor is that ISTs have been proposed as a treatment for the inflammatory phase of COVID-19 [19]. Given these assumptions, it is difficult to understand whether immunosuppression should be considered a risk factor for a bad prognosis or a possible treatment strategy, and its management in COVID-19 patients is yet to be established.

Among the immunosuppressive drugs used for solid organ transplantation [20] and for systemic rheumatic disorders (RMDs) [21], we focused our attention on two calcineurin inhibitors (CNIs): cyclosporine A (Cys) and tacrolimus (TAC). These drugs might exert an antiviral activity through different mechanisms, as evidenced in vitro on some coronavirus strains [22,23,24,25] and HIV [26]. We report herein our observations obtained from the adoption of a new protocol developed for the management of patients on CNI maintenance therapy and infected with SARS-CoV-2. The aim of the study was to evaluate the frequency, the clinical manifestations, and the outcome of SARS-CoV-2 infection in this population during the outbreak of COVID-19 in Italy.

## 2. Materials and Methods

In February 2020, our multidisciplinary team defined the protocol to be applied in COVID-19 patients undergoing ISTs and followed in the divisions of Rheumatology, Respiratory Diseases, Nephrology, Cardiology, and Cardiac Surgery at our hospital. The cohort included patients followed-up for RMDs such as antisynthetase syndrome (ASSD), idiopathic inflammatory myopathies (IIMs), systemic lupus erythematosus (SLE), or adult-onset still disease (AOSD), and for solid organ transplantation (heart, lung, and kidney). The protocol was aimed at homogenizing the management of immunosuppressive treatment at our institution, in order to minimize the risk of both disease flare/organ rejection and COVID-19 severe manifestations. A consensus decision was reached between expert clinicians in IST management (LC, FM, MG, CP), consisting in: (1) the maintenance of Cys and TAC without dose reduction; (2) the tapering of 50% of the dosage for drugs such as mycophenolate mofetil and azathioprine; (3) the withdrawal of everolimus, methotrexate, and other not listed immunosuppressants; (4) the maintenance of corticosteroid dosage.

Subsequently, all patients in the cohort, recruited from the areas with the highest incidence of COVID-19 in Italy (Lombardy, Emilia Romagna, Piedmont, Veneto), were strictly followed with weekly phone contact, aiming at the early identification of SARS-CoV-2 infection. The study was approved by the IRB of the IRCCS Policlinico S. Matteo Foundation of Pavia (protocol number 202200053246), and all patients agreed to participate in this interview and provided informed consent. Patients with clinically suspected COVID-19 were referred for a rhino-pharyngeal swab for SARS-CoV-2. In the case of a positive swab, they were diagnosed with COVID-19 and selected for the study. In our hospital, rhino-pharyngeal swabs were performed by RT-PCR [27].

Patients with COVID-19 admitted to our hospital were assessed directly, whereas the others were assessed by phone contact. In COVID-19 patients, we investigated the occurrence and duration of typical symptoms, ongoing treatments, hospital admission, and, if possible, laboratory and chest X-ray results. Patients were considered as having lung involvement in the case of bilateral pneumonitis at chest X-ray, or new appearance/worsening of dyspnea with significant lowering of peripheral oxygen saturation (SaO_2_ < 92%). The outcomes were also identified: full recovery, improvement (partial relief from symptoms), no improvement (symptoms unchanged), worsening (onset of new symptoms or worsening of existing ones), and death. Collected data were updated weekly until 28 April 2020, more than 2 months after the beginning of the COVID-19 outbreak in Italy.

### Statistical Analysis

Data for categorical variables have been reported as absolute numbers and percentages. Median and interquartile range have been used to describe numerical variables. Chi-square or Fisher exact tests were applied to explore differential distributions among the different groups of patients. COVID-19 occurrence was expressed as a relative frequency (number of cases at the end of the survey divided by the total number of patients), by type of patient and in the whole sample of patients treated with CNIs. A *p*-value of less than 0.05 was considered statistically significant. The analyses were performed by one statistician (MCM) and one healthcare professional experienced in statistical analysis (LC) using Stata 15 (StataCorp. 2017. Stata Statistical Software: Release 15. College Station, TX, USA: StataCorp LLC.).

## 3. Results

### 3.1. Baseline Characteristics of the Cohort

We identified 385 patients eligible for the study (220 males, 57%; 165 females, 43%; TAC 214, 56%; Cys 171, 44%) and collected data from each patient during the pandemic period. Most patients were solid organ transplantation recipients (kidney *n* = 140, 36%; heart *n* = 100, 26%; lung *n* = 91, 24%), while 54 patients (14%) suffered from RMDs: 42 patients had ASSD with interstitial lung disease (ILD) (78% of RMDs), 2 had IIMs (4%), 7 had SLE (12%), and 3 had AOSD (6%). The overall characteristics of the patients enrolled in the study are reported in Table 1.

Forty-seven patients (12%) were treated exclusively with CNIs (TAC *n* = 10, 12%; Cys *n* = 37, 78%) and 338 (88%) were also treated with other ISTs in multiple association (corticosteroids *n* = 203, 53%; mycophenolate mofetil *n* = 188, 49%; everolimus *n* = 65, 17%; azathioprine *n* = 17, 4%; other *n* = 23, 6%). Blood levels of Cys and TAC were found to be in the therapeutic range in all cases, according to the underlying condition (rheumatic diseases, treated only with Cys, range between 100 and 150 ng/mL) and the type of and time from transplantation (different ranges used for Cys and TAC) for each patient. The drugs’ circulating levels were obtained during hospitalization in COVID-19 patients, and from a clinical chart review in patients without COVID-19.

### 3.2. COVID-19 Patient Characteristics and Outcomes

A diagnosis of COVID-19 was reached in 14 cases (4%), all of which were transplant recipients. Only two patients (14%) were transplanted less than 1 year ago.

In all COVID-19 cases, we applied the developed protocol. We did not observe a gender prevalence (10 males and 4 females, *p* = 0.276) nor any discrepancy in infection rate between patients on combination therapy vs patients on CNIs alone (3 vs. 11 cases, *p* = 0.267). Patients more frequently reported fever (12 cases, 86%), diarrhea (10 cases, 71%), and fatigue (10 cases, 71%), whereas dyspnea was observed in only three cases (21%). The complete list of symptoms observed in our cohort of COVID-19 patients is reported in Table 2.

COVID-19-related lung involvement was observed in eight cases (57% of COVID-19 patients), symptomatic in three cases, and, only detected by X-ray in five cases. None of the patients developed severe respiratory complications, including the 42 (11% of the total cohort) RMD patients with interstitial lung disease (ILD) and the 91 (24%) lung-transplanted patients. Circulating levels of Cys and TAC were in all cases in the therapeutic range, which was different according to the different times from transplantation.

The median symptom duration was 13.5 days (IQR 10-15). The outcome was favorable in most cases: 12 patients (86%) fully recovered. Two (14%) patients died, one (male, 68 years old, heart-transplanted in 2006) 12 days after symptom onset, and the other one (female, 67 years old, heart-transplanted in 2004) after 8 days. Of note, the first patient suffered from metastatic lung cancer and was in palliative care, while the second one had chronic cardiac rejection, multivasal coronopathy with multiple stenting (the last in December 2019), and a grade IV chronic kidney disease. Twelve COVID-19 patients (86%) were hospitalized, mainly for fever and diarrhea, which were in all cases SARS-CoV-2-related. Only the 67 year-old woman who died was admitted for fever and dyspnea due to SARS-CoV-2-related pneumonia. Just one (male, 71 years old, kidney transplant in 2015) of the remaining 10 patients was re-admitted 15 days after his first discharge for the occurrence of a pulmonary embolism while he was on apixaban. He was treated with heparin (100 UI/kg twice daily) and now he has fully recovered. All immunosuppressed COVID-19 patients started hydroxychloroquine (HCQ) (600 mg for the first day of treatment followed by 400 mg for 10 days), azithromycin (500 mg daily for 7 days), and heparin (100 UI/kg once daily), following our internal guidelines at that specific period. No superimposed infections were observed in our cohort. Only the second reported patient that died required initiation of high-flow O2 therapy a few hours before death. No patient developed ARDS. Characteristics of patients with a diagnosis of COVID-19 are summarized in Table 3.

## 4. Discussion

The effect of SARS-CoV-2 infection on patients who are on immunosuppressive treatment is still a matter of debate. Although the frequency of COVID-19 was quite high in our cohort (4%), the clinical presentation was generally mild and no patient progressed to ARDS. A possible explanation for this increased frequency of COVID-19 could be the stringent controls we applied to these patients, who underwent rhino-pharyngeal swabs earlier than the general population, notwithstanding the mild presentation of symptoms.

Another point to highlight is that the outcome of our patients was generally favorable. In fact, the clinical condition of almost all patients rapidly improved. Only two patients had a negative outcome: in both cases, it was only partially attributable to SARS-CoV-2 infection, considering the strongly compromised pre-COVID-19 conditions. We observed only one confirmed thrombotic complication, which occurred after the acute phase of the disease, in the sole patient in which heparin was not administered due to ongoing treatment with apixaban. Interestingly, no patients developed superimposed infections despite the maintenance of CNIs, and no transplanted patients had organ rejection after the withdrawal of non-CNI immunosuppressants. We observed COVID-19 cases only in the setting of transplantation and not in RMDs, although the lower number of patients in the latter group might explain this finding. Different from what has been reported in other cohorts/cases [13,14,15,16,17,18], the outcome of our transplanted patients was substantially favorable. The surprisingly low severity of COVID-19 infection in our cohort might have three possible explanations. As previously reported, the rigorous controls we adopted may have facilitated the early diagnosis, hospitalization, and treatment of these patients. The long-term immunosuppressive regimen used in our cohort might have influenced the clinical course of the disease by preventing the occurrence of huge alveolar macrophage activation with consequent release of pro-inflammatory cytokines described in the context of COVID-19 [19]. The continuation of CNI treatment we adopted could explain, at least in part, the better outcome of our patients compared to other cohorts/case series [14,15,16,17,18] in which CNIs were interrupted at COVID-19 diagnosis. However, in a paper from the United States [13] showing a high frequency of ARDS (11 out of 36 patients, 39%) and high mortality in a group of kidney-transplanted COVID-19 patients (10 out of 36 patients, 28%), the therapeutic approach to ISTs was similar to ours, observing the withdrawal of TAC in only six cases. We do not know if these six patients survived. Furthermore, besides ethnicity, the only concrete difference we observed is that the US patients were treated with apixaban, whereas in our cohort we used heparin in all but one patient who developed pulmonary embolism. Considering some previous warnings about the use of apixaban on COVID-19 patients [28], we can hypothesize that this difference could have influenced, at least partially, the evolution of the disease. Finally, we cannot exclude a direct antiviral activity of CNIs. Both Cys and TAC inhibit viral replication in many strains of CoV, including SARS-CoV, through the inhibition of peptidyl-prolyl cis-trans isomerases, such as cyclophilin A and FK506-binding proteins, which are cellular interaction partners of CoV non-structural protein 1 (Nsp1) [22,23,24]. If we hypothesize that Cys and TAC also exert antiviral activity towards SARS-CoV-2, we may presume an added value of CNIs with respect to other immunosuppressants, because they are theoretically able to reduce the viral load or the risk of viral load increase. On this basis, it is reasonable to add CNIs to the list of ISTs as possible therapeutic options for COVID-19 [29], although no studies on CNIs’ action on SARS-CoV-2 replication are currently available.

Our study has some limitations. First, we cannot exclude the possibility that some patients could develop the disease in the next few days or weeks, but this limitation is common to every cohort study on COVID-19 published thus far. Second, we included patients with different underlying conditions, such as solid organ transplantation and RMDs. However, the purpose of our study was to evaluate the effect of CNIs on the course of COVID-19 rather than the outcome of different subsets of patients. Third, our study lacked a matched control group. As previously explained, during the period of our observation, non-immunosuppressed COVID-19 patients were generally admitted to our hospital with a more compromised respiratory picture as compared to our patients (mostly admitted for SARS-CoV2-related fever and diarrhea, which should be carefully considered as COVID-19 warning signs in these patients), thus making it difficult to identify a control group. Therefore, we decided not to include a comparison of circulating drug levels between COVID-19 and non-infected patients, because they were collected at different time points since it was only possible to test hospitalized patients during the lockdown.

This preliminary report suggests that CNIs do not negatively affect the course of COVID-19, not even in patients with SARS-CoV-2-related lung involvement, and therefore they should not be discontinued. Furthermore, it is important to underline that no patient developed infectious complications. The substantial lack of ARDS in our cohort may also suggest a protective action of immunosuppression in these patients, at least for one of the most dreadful complications of the disease. This might be especially true in the case of CNIs, which, in addition to their action on the immune system, could also exert an antiviral action on coronavirus. In conclusion, our result indicates the need for a careful discussion about the effect of immunosuppression on COVID-19.

## Figures and Tables

**Table 1:** General characteristics of patients evaluated.

	Patients (%)	Median Age in Years (IQR)	Median Follow-Up in Months (IQR)	Males (% by Group)	Cys (% by Group)	TAC (% by Group)	CNIs and Other ISTs (% by Group)	COVID-19 (%)	Median Age in Years (IQR)	Median Follow-Up in Months (IQR)	Males (% by Group)	Cys (% by Group)	TAC (% by Group)	CNIs and Other ISTs (% by Group)
RMDs	53 (14)	61 (48-69)	41 (17–81)	15 (28)	53 (100)	0 (0)	30 (57)	0 (0)	-	-	-	-	-	-
Lung Tx	91 (24)	58 (48-64)	116 (46–187)	54 (59)	11 (12)	80 (88)	91 (100)	3 (21)	65 (62–75)	60 (1–266)	2 (67)	1 (33)	2 (67)	3 (100)
Heart Tx	100 (26)	63 (53-72.5)	144 (72–228)	61 (61)	63 (63)	37 (37)	78 (78)	5 (36)	63 (53–67)	60 (48–144)	3 (60)	2 (40)	3 (21)	3 (60)
Kidney Tx	140 (36)	55 (48-62.5)	62.5 (32.5–116.5)	90 (64)	43 (31)	97 (69)	139 (99)	6 (43)	57.5 (51–64)	95 (46–159)	5 (83)	3 (50)	3 (21)	5 (83)
Overall	384 (100)	58 (49-66)	83.5 (39–156)	220 (57)	170 (44)	214 (56)	338 (88)	14 (100)	60.5 (46–159)	62.5 (53–67)	10 (71)	6 (43)	8 (57)	11 (79)

COVID-19: coronavirus disease 2019; IQR: interquartile range; Tx: transplantation; Cys: cyclosporine; TAC: tacrolimus; RMDs: rheumatic diseases; ISTs: immunosuppressive treatments.

**Table 2:** Symptom prevalence in the COVID-19 cohort (COVID-19 = 14).

Symptoms	COVID-19, Number of Patients (%)
Fever	12 (86)
Fatigue	10 (71)
Cough	9 (64)
Rhinorrhea	5 (35)
Headache	8 (57)
Diarrhea	10 (71)
Nausea	8 (57)
Sore throat	4 (28)
Ageusia	4 (28)
Arthromyalgias	4 (28)
Dyspnea	3 (21)
Anosmia	5 (36)
Conjunctivitis	2 (14)

**Table 3:** Characteristics of the 14 patients with confirmed COVID-19.

Condition (Number, % of Overall)	Median Time from Symptom Onset in Days (IQR)	Hospitalization (% of the Group)	Clinical/Radiological Lung Involvement (% of the Group)	ARDS (% of the Group)	Full Recovery (% of the Group)	Improved (% of the Group)	Stable (% of the Group)	Worsened (% of the Group)	Death (% of the Group)
RMDs (0, 0)	-	-	-	-	-	-	-	-	-
Lung Tx (3, 21)	15 (10-18)	2 (67)	2 (67)	0 (0)	3 (100)	0 (0)	0 (0)	0 (0)	0 (0)
Heart Tx (5, 36)	12 (10-15)	5 (100)	2(40)	0 (0)	3 (60)	0 (0)	0 (0)	0 (0)	2 (40)
Kidney Tx (6, 43)	12.5 (10-15)	5 (80)	5 (83)	0 (0)	5 (80)	1 (20)	0 (0)	0 (0)	0 (0)
Overall (14, 100)	13.5 (10-15)	12 (86)	9 (64)	0 (0)	11 (79)	1 (7)	0 (0)	0 (0)	2 (14)

COVID-19: coronavirus disease 2019; RMDs: rheumatic diseases; Tx: transplantation; ARDS: acute respiratory distress syndrome.
